# Artificial intelligence in diagnosis of pediatric neurodevelopmental disorders: a scoping review

**DOI:** 10.1007/s12519-025-00999-z

**Published:** 2026-01-27

**Authors:** María Alejandra Nieto Ramírez, Mateo Mariño Rodríguez, María José Castro Salas, Erwin Hernando Hernández Rincón

**Affiliations:** 1 School of Medicine, Universidad de La Sabana, Chía, Colombia; 2 Department of Family Medicine and Public Health, School of Medicine, Universidad de La Sabana, Campus del Puente del Común, km. 7, Autopista Norte de Bogotá, Chía, Colombia

**Keywords:** Artificial intelligence, Biosignals, Diagnosis, Machine learning, Neurodevelopmental disorders, Pediatrics

## Abstract

**Background:**

Neurodevelopmental disorders are a group of conditions that affect key areas of development and may significantly impact a child’s quality of life. This underscores the importance of accurate diagnostic tools to improve outcomes. Artificial intelligence (AI) has shown measurable effectiveness for enhancing the diagnosis and monitoring of neurodevelopmental disorders. This scoping review aims to summarize the current evidence on the use of AI technologies, including deep learning, supervised machine learning, decision support systems, and biosignal analysis, in improving diagnostic accuracy for pediatric neurodevelopmental disorders.

**Data sources:**

A systematic search was conducted across PubMed, LILACS, MEDLINE, Google Scholar, and psychology-indexed journals, covering publications from 2000 to January 2025. Keywords and Medical Subject Headings terms were used to search for and select studies, applying specific inclusion and exclusion criteria. Selection followed the Preferred Reporting Items for Systematic Reviews and Meta-Analyses extension for Scoping Reviews guidelines and included clinical studies, reviews, and validation research. The data were extracted and synthesized descriptively.

**Results:**

Twenty-two studies were included. Deep learning models achieved diagnostic accuracies exceeding 85% in most studies in neuroimaging interpretation, whereas supervised machine learning improved the subtype classification of autism spectrum disorder and attention deficit hyperactivity disorder. Decision support systems have increased diagnostic efficiency, and biosignal-based AI has shown potential in identifying physiological markers related to neurodevelopmental disorders.

**Conclusions:**

AI technologies may significantly contribute to improving early diagnosis and clinical decision-making in pediatric neurodevelopment. However, variability in study design, population, and algorithm standardization remains a challenge. AI technologies are also facing ethical concerns such as data privacy and security, interpretability, equity and access, and algorithmic bias. Further multicenter validation and regulatory frameworks are essential for clinical translation.

**Graphical abstract:**

**Supplementary Information:**

The online version contains supplementary material available at 10.1007/s12519-025-00999-z.

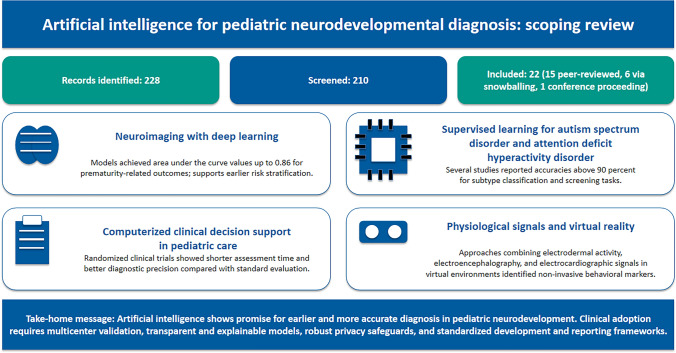

## Introduction

Neurodevelopmental disorders (NDDs) are a group of conditions that impact key areas, such as cognition, language, behavior, and motor function, significantly affecting quality of life, academic performance, and social interaction if timely diagnosis and treatment are not obtained. These disorders include autism spectrum disorder (ASD), attention deficit hyperactivity disorder (ADHD), and intellectual disability. Globally, about 20% of children are estimated to have mental disorders, with ASD being one of the leading causes of disability in childhood and adolescence [1, 2].

According to figures published periodically by the U.S. Environmental Protection Agency, the most prevalent diagnoses among 5- to 17-year-olds include ADHD (10.7%, predominantly in boys), ASD (2.8%), specific learning disorders (SpLDs) (8.8%), and developmental coordination disorders (DCDs) (5%–6% in school-aged children) [3]. In addition, a study in the rural population of Oaxaca reported a prevalence of 43% specific developmental disorders (SDD), with a predominance of males, affecting mainly the area of language (29%), followed by gross motor skills [4]. In Colombia, information on the prevalence of NDD is limited and comes from specific studies. An analysis in Medellín between 2009 and 2012 reported that the main diagnoses in children seen in a neuropsychology unit were ADHD (39.3%), mild mental retardation (5%), and mixed anxiety and depressive disorder (3.8%) [5]. The diagnosis of NDD represents a considerable challenge owing to variability in clinical presentation, subjectivity in assessment, and reliance on conventional tools such as clinical scales, behavioral observation, and structured interviews. These methods, although widely used, have limitations in terms of accuracy and reproducibility, which can lead to late or erroneous diagnoses, affecting timely access to appropriate therapeutic interventions [6].

In this context, artificial intelligence (AI) has emerged as a promising tool to improve diagnostic accuracy in individuals with NDDs. AI comprises a set of advanced technologies with the ability to perform tasks such as learning, decision-making, and pattern recognition. Its application in this field has proven to be effective in the analysis of clinical data, neuroimaging, digital biomarkers, and behavioral patterns, optimizing early detection and reducing diagnostic variability. Among its main advantages are the ability to detect subtle patterns in complex data, the reduction in diagnostic variability between clinicians, and the possibility of customizing assessments to the individual characteristics of each patient. However, despite the growing interest in its use, evidence is still developing. Although multiple studies are exploring its application, important challenges remain, such as the lack of validation in real clinical settings and its integration into health systems, which has limited its implementation in clinical practice and generated the need for additional research to evaluate its efficacy and applicability [7].

Based on the above, the main objective of the present research is to map the literature on the use of AI in the diagnostic accuracy of children at risk or suspected of NDD, identifying the most used technologies and their effectiveness. This analysis provides an understanding of the current state of knowledge in the area, identifies research gaps, and provides evidence to facilitate the development and implementation of AI-based tools in clinical practice.

## Methods

This scoping review was registered and managed in the Open Science Framework (OSF) under the identifier OSF.IO/JXH5Y. The protocol followed a structured methodological framework to address the research question: “What is the available evidence on the use of AI to improve diagnostic accuracy in children at risk of, or suspected of having, NDDs?”

To guide the review, the Joanna Briggs Institute (JBI) methodology for scoping reviews was adopted. Study identification and selection were reported in accordance with the Preferred Reporting Items for Systematic Reviews and Meta-Analyses extension for Scoping Reviews (PRISMA-ScR) checklist.

**Fig. 1:**
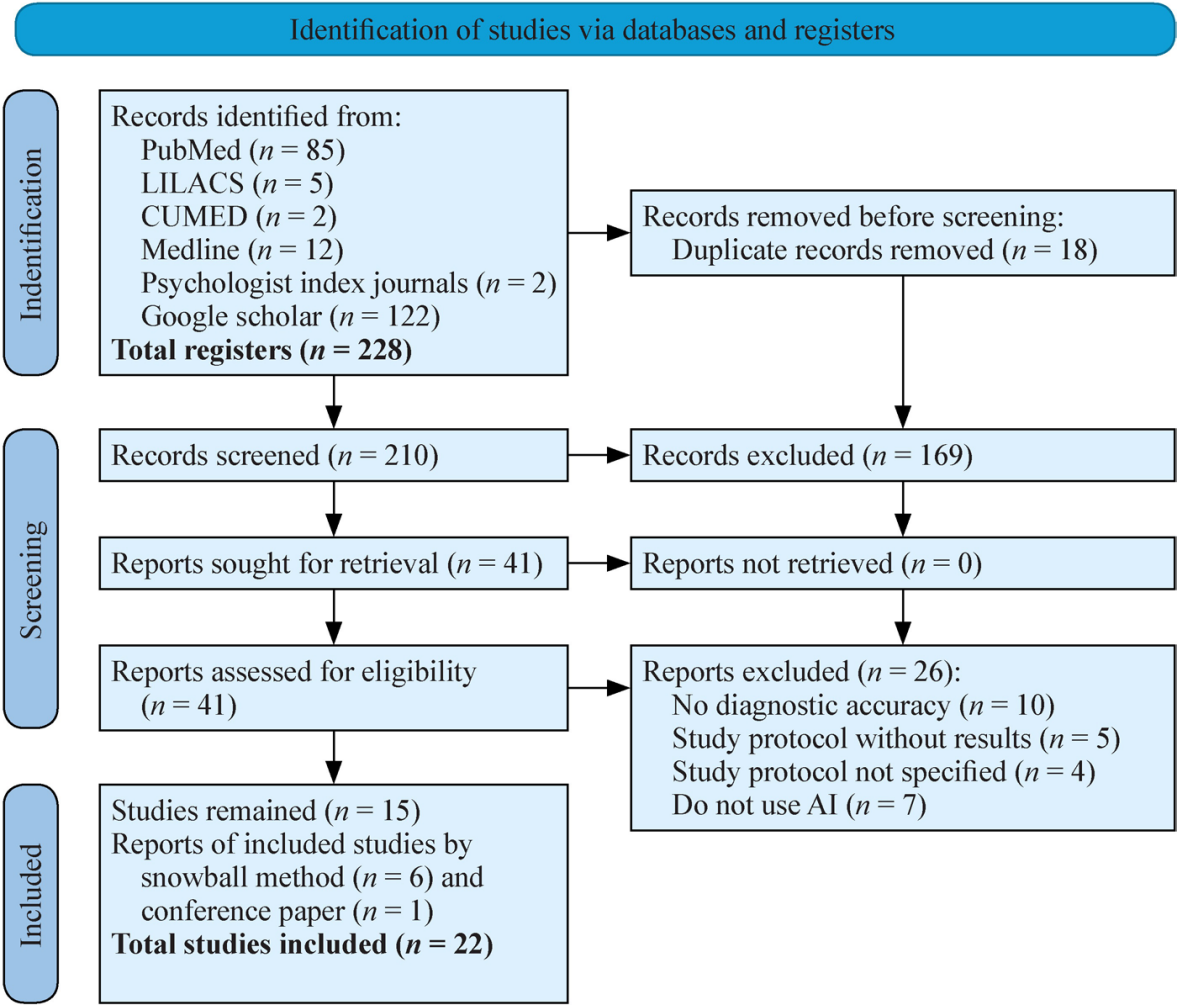
Identification of studies in PRISMA format

A comprehensive search strategy was developed to minimize selection bias, using Medical Subject Headings (MeSH) terms and Boolean operators across multiple databases: PubMed, MEDLINE, LILACS, CUMED, Google Scholar, the Virtual Health Library (VHL), and psychology-focused journals. The final search was conducted in January 2025, and duplicate records were removed prior to screening. The initial search yielded 228 records, 210 of which were assessed for relevance. Fifteen studies met the inclusion criteria, and an additional six were identified through reference searching (“snowballing:), resulting in a total of 22 studies included in the review (Fig. 1 and Supplementary Table). All records were managed via Rayyan software.

The selection process was carried out by three independent reviewers. Two reviewers (MANR and MMR) independently screened titles and abstracts. Discrepancies were resolved through discussion or by a third reviewer (MJCS), who acted as a tiebreaker. The full texts of the eligible studies were assessed in duplicate. All reviewers received training in scoping review methodology, and predefined inclusion and exclusion criteria were applied consistently.

Studies were eligible if they were open access, published in English or Spanish between January 2000 and January 2025, and focused on the use of AI techniques such as machine learning, neural networks, or decision support systems for diagnosing NDDs in pediatric populations (Table 1). This timeframe allowed for the analysis of research evolution, as well as the identification of emerging trends, technological advances, and methodological developments. Studies also had to report diagnostic accuracy metrics [e.g., sensitivity, specificity, area under the curve (AUC)-receiver operating characteristic (ROC)] or evaluate the impact of AI on early detection and access to interventions.

**Table 1:** Distribution of included studies by artificial intelligence methodology and neurological disorder subtype

AI methodology	Number of studies	Main diagnoses	Example references
Deep neural networks	6	Prematurity, ASD	[8–13]
Supervised machine learning (SVM, random forest, etc.)	10	ASD, ADHD	[11–20]
Clinical decision support systems	3	ADHD, developmental delay	[21–23]
Biosignal analysis + VR/EDA/EEG	4	ASD	[11, 23–25]

*ASD* autism spectrum disorder, *ADHD* attention deficit hyperactivity disorder, *SVM* support vector machine, *VR* virtual reality, *EDA* electrodermal activity, *EEG* electroencephalogram

Clinical trials, observational studies, systematic reviews, meta-analyses, and AI validation studies were included. Gray literature was excluded because of the absence of peer review, limited methodological transparency, indexing difficulties, and its potential to introduce information bias, which could compromise the quality, reliability, and reproducibility of the findings. Although grey literature was generally excluded, we included one conference proceeding (Reyes et al., 26) as an exception because it reported an AI-based diagnostic approach in a pediatric population with adequate methodological detail and relevance

Data, including the lead author, title, year of publication, study design, interventions, main diagnoses, outcomes, and AI approach used, were extracted from each study (Table 2). These elements were selected to provide a comprehensive overview of the research landscape of the role of AI in improving diagnostic accuracy in neurodevelopmental disorders.

**Table 2:** Article summary

Number	Authors	Title	Year	Design	Diagnosis	AI used	Intervention
1	He et al. [8]	A multitask, multistage deep transfer learning model for early prediction of neurodevelopment in very preterm infants	2020	Retrospective observational	Preterm infants with neurodevelopmental deficits in cognitive, language, motor, cognitive, and motor areas	Deep learning, transfer learning, multitask learning, and multimodal data fusion—AI in computational neuroscience	Development and validation of a multitask, multistage deep learning model for early prediction of neurodevelopment in premature infants. Utilized brain connectome and clinical data
2	Saha et al. [9]	Predicting motor outcome in preterm infants from very early brain diffusion MRI using a deep learning CNN model	2020	Predictive model validation study	Premature infants at high risk of motor neurodevelopmental deficits	Deep learning with CNNs-computer applied to clinical neuroscience	Use of a CNN-based deep learning model for prediction of motor outcomes from early brain diffusion magnetic resonance imaging
3	Qureshi et al. [14]	Multiclass classification for the differential diagnosis on the ADHD subtypes using recursive feature elimination and hierarchical extreme learning machine: structural MRI study	2016	Observational study	ADHD	Supervised machine learning with ELM and feature selection (RFE)-brain neuroimaging	Application of a H-ELM combined with RFE-SVM to analyze structural magnetic resonance images. It allowed the classification of three groups: typical development, inattentive type, and combined type
4	Andrade et al. [15]	A protocol for the diagnosis of autism spectrum disorder structured in machine learning and verbal decision analysis	2021	Quantitative and qualitative: hybrid protocol	ASD	Machine learning, supervised models, and analysis based on natural language or fuzzy logic	Application of hybrid model (integration of random forest algorithm and ZAPROS-III method) for verbal decision analysis to identify key features for the diagnosis of ASD
5	Heinsfeld et al. [10]	Identification of autism spectrum disorder using deep learning and the ABIDE dataset	2017	Quantitative and observational	ASD	Deep learning (CNNs or RNNs) and neuroimaging analysis	Application of supervised and unsupervised machine learning methods to analyze brain functional connectivity patterns and classify participants according to the presence of ASD
6	Raya et al. [11]	Application of supervised machine learning for behavioral biomarkers of autism spectrum disorder based on electrodermal activity and virtual reality	2020	Clinical trial	ASD	Machine learning applied in EDA and VR	A VR environment was used to simulate social and emotional situations to capture electrodermal activity responses. The data obtained were processed using supervised learning algorithms to identify behavioral biomarkers characteristic of ASD
7	Ismail E et al. [12]	HEC-ASD: a hybrid ensemble-based classification model for predicting autism spectrum disorder disease genes	2022	Model development and validation	ASD	Gradient boosting ensemble (HEC-ASD) with gene ontology similarity	Created gene‒gene functional similarity matrices using gene ontology, then applied a gradient boosting ensemble classifier to predict ASD genes in SFARI data
8	Ismail E et al. [13]	A hybrid stacking SMOTE model for optimizing the prediction of autistic genes	2023	Model development and validation	ASD	Stacking SMOTE ensemble (SMOTE + GBBRF + RF + SVM + k-NN + LR)	Applied SMOTE for balancing gene-class data, used gene ontology to compute gene similarity, then built a stacked ensemble classifier combining multiple algorithms to predict ASD genes
9	Carroll et al. [21]	Use of a computerized decision aid for ADHD diagnosis: a randomized controlled trial	2013	Randomized clinical trial	ADHD	Machine learning or an expert system	Application of two tools in two groups (computerized diagnostic aid tool and standard diagnostic method). The tool provided algorithm-based support to improve diagnostic accuracy and efficiency
10	Carroll et al. [22]	Use of a computerized decision aid for developmental surveillance and screening	2014	Randomized clinical trial	Neurodevelopmental delay	Machine learning or rule-based systems	Application of a computerized tool to guide developmental assessment (integration of algorithms to improve detection of developmental delays) compared to a group using the standard method without digital support
11	Alcañiz et al. [23]	Biomarkers of autism spectrum disorder based on biosignals, virtual reality, and artificial intelligence	2020	Narrative review	ASD	Machine learning applied to EEG, EDA, heart rate, etc., and VR as an evaluation tool (Biosignals)	Use of VR to simulate social interactions while measuring biosignals such as electroencephalogram and electrodermal activity. The data were processed by artificial intelligence algorithms where specific patterns were identified that could serve as behavioral biomarkers of ASD
12	Reyes et al. [26]	Application of artificial intelligence to presumptive diagnosis of the following diseases neurodevelopment in at-risk infants	2023	Observational and based on artificial intelligence	Neurodevelopmental disorders	Machine learning	Implementation of an AI system for analysis of clinical data and developmental patterns in at-risk children to detect early signs of neurodevelopmental impairment and support clinical decision-making
13	Washington et al. [27]	Data-driven diagnostics and the potential of mobile artificial intelligence for digital therapeutic phenotyping in computational psychiatry	2021	Narrative review	ASD	Eye tracking and computer vision	Use of digital technologies such as eye tracking and computer vision for autism screening, as well as digital therapeutic interventions that allow for data capture and longitudinal tracking of outcomes as diagnosis progresses
14	Abbas et al. [28]	Machine learning approach for early detection of autism by combining questionnaire and home video screening	2018	Clinical validation study in a controlled clinical trial	ASD	Machine learning	Two machine learning algorithms for autism detection; one based on structured questionnaires for parents and the other on the analysis of key behaviors in short home videos. In addition, a combined algorithm improves diagnostic accuracy
15	Welch et al. [29]	Use of mobile and wearable artificial intelligence in child and adolescent psychiatry: scoping review	2022	Scoping review	ASD, ADHD, internalizing disorders	Biosensors	Use of biosensors in 19 studies using cardio-fitness bands with ECG sensors for heart rate monitoring and wrist biosensors, such as Fitbit watches, for measuring physiological variables
16	Fernandez et al. [30]	Technological tools for the diagnosis and treatment of attention deficit hyperactivity disorder	2020	Narrative review	ADHD	Machine learning, neural networks, or rule-based models	Analysis of technological tools used for the diagnosis and treatment of ADHD (DIDE, MOXO, AULA, AQUARIUM, BRAINAGAZE) and therapies based on neurofeedback, transcutaneous electrical stimulation of the trigeminal nerve and applications such as SINCROLAB and PSIOUS
17	Shahamiri et al. [19]	A new classification system for autism based on machine learning of artificial intelligence	2021	Observational and machine learning-based	ASD	Machine learning	Application of machine learning algorithms for analysis of clinical and neuroimaging data to identify patterns that allow a more accurate classification of ASD
18	Ghafghazi et al. [24]	AI-augmented behavior analysis for children with developmental disabilities: building toward precision treatment	2021	Exploratory and artificial intelligence-based study	ASD	Deep learning and behavioral analysis	An AI-assisted behavioral analysis system was used, which collects and analyzes data on behavioral patterns in children with developmental disorders
19	Minissi et al. [25]	Assessment of the autism spectrum disorder based on machine learning and social visual attention: a systematic review	2021	Systematic review	ASD	Machine learning in visual attention analysis	Compiled and analyzed research that used machine learning algorithms in conjunction with data on social visual attention patterns in children with ASD. Evaluated how these technologies can improve accuracy in the detection of the disorder
20	Quintero et al. [20]	Predicting ADHD with machine learning: systematic literature review	2024	Systematic literature review	ADHD	Machine learning, neural networks, SVMs, and decision trees applied to neuroimaging	A literature search in which they analyzed different machine learning algorithms applied to the diagnosis of ADHD by means of psychometric tests, electroencephalograms, brain imaging, and motion analysis
21	Bölte et al. [31]	How can clinicians detect and treat autism early? Methodological trends of technology use in research	2015	Narrative review	ASD	Machine learning, deep learning, expert systems, and biosignal analysis	Analysis of previous studies exploring the use of technologies such as AI, VR, and biosignals in the early detection and treatment of ASD. Methodological trends in the technological approaches employed are evaluated
22	Hyde et al. [32]	Applications of supervised machine learning in autism spectrum disorder research: a review	2019	Literature review	ASD	Machine learning—neural networks, SVMs, decision trees	It reviews several studies that apply supervised machine learning, such as SVMs, neural networks, and decision trees, to analyze biomarkers of ASD

*ASD* autism spectrum disorder, *ADHD* attention deficit hyperactivity disorder, *AI* artificial intelligence, *ROC* receiver operating characteristic curve, *EEG* electroencephalogram, *ECG* electrocardiogram, *EDA* electrodermal activity, *AI* artificial intelligence, *VR* virtual reality, *ELM* extreme learning machine, *H-ELM* hierarchical extreme learning machine, *RFE* recursive feature elimination, *SVM* support vector machine, *CNN* convolutional neural network, *RNN* recurrent neural network, *SMOTE* synthetic minority oversampling technique, *GBBRF* gradient boosted balanced random forest, *RF* random forest, *k-NN* k-nearest neighbors, *LR* logistic regression, *HEC-ASD* hybrid ensemble-based classification model for autism spectrum disorder, *MRI* magnetic resonance imaging, *ZAPROS-III* method of pairwise comparison of alternatives by successive concession (ЗAПPOC-III in Russian), *ABIDE* autism brain imaging data exchange, *HEC-ASD* hybrid ensemble-based classification ASD, *SFARI* Simon’s foundation autism research initiative, *DIDE* diagnostic interview for attention deficit hyperactivity disorder, MOXO continuous performance test, *AULA* Aula Nesplora (a virtual reality test in a simulated classroom for attention and impulsivity assessment), *AQUARIUM* Nesplora aquarium (virtual reality test designed for the evaluation of executive functions and attention), *BRAINAGAZE* brain and gaze interaction technology, *SINCROLAB* Sincrolab cognitive training platform, *PSIOUS* Psious virtual reality platform (virtual reality platform used for clinical psychological therapies)

The lead author and title helped identify the source and scope of each study. The year of publication allowed for the analysis of temporal trends. The study design contributed to the assessment of methodological quality. The interventions and diagnoses described how AI was applied in clinical settings and its role in early detection. The reported outcomes supported the synthesis of key findings relevant to pediatric clinical practice. The AI approach provides insight into the specific algorithms, techniques, and models used, allowing for the identification of patterns and the evaluation of their effectiveness.

Finally, data synthesis was conducted via descriptive analysis to identify the main applications of AI in enhancing diagnostic accuracy for children at risk of, or suspected of having, neurodevelopmental disorders.

## Results

After the search strategy was applied to multiple databases and information sources, a total of 228 records were identified. After removing duplicates (*n* = 18), 210 records were screened and 169 were excluded. Then 41 full-text reports were assessed for eligibility, of which 26 were excluded. Fifteen studies met the inclusion criteria; six additional studies were identified through snowballing, and one conference proceeding (gray literature) was also included, for a total of twenty-two studies. The studies included in this review explored various applications of artificial intelligence in the diagnosis, classification, and treatment of neurodevelopmental disorders. Overall, four main approaches of AI used were identified: (1) deep neural networks (deep learning) and diagnostic accuracy of NDD; (2) supervised machine learning and diagnosis of ASD and ADHD; (3) computerized decision aids and prediction of NDD, and (4) biosignal analysis for early diagnosis of NDD and virtual reality (AI-enhanced biomarkers). Some of these results are summarized in Table 3.

**Table 3:** Results summary

Study	IA model used	Diagnosis	Precision	Specificity/sensibility	AUC	Key data	Interpretation
He et al. [8]	Deep transfer learning (DNN)	Prematurity	81.5%	–	0.86	–	Employing such a deep learning model may facilitate risk stratification at term-equivalent age for early identification of long-term neurodevelopmental deficits and targeted early interventions to improve clinical outcomes in very preterm infants
Saha et al. [9]	Deep learning (CNN)	Motor deficits	–	Sensitivity: 70%, Specificity: 74%	0.72	–	Moderate precision in motor abnormalities detection
Qureshi et al. [14]	RFE + H-ELM (supervised ML)	ADHD	60.78%–85.29%	–	–	–	High precision in binary combination, whereas it decreases in multiclass classification for ADHD classification
Heinsfeld et al. [10]	SVM, Random Forest, DNN	ASD	63%–70%	Sensitivity: 68%–74%, specificity: 58%–63%	–	–	Significatant precision in identification of patients with ASD mostly with DNN
Raya et al. [11]	Supervised ML + VR/EDA	ASD	84%–90%	–	–	–	High accuracy on classification of physiological response which reveals that the implementation of this tool could be useful for early diagnosis in ASD
Ismail et al. [12]	Gradient boosting + gene ontology	ASD (genetic high biomarkers)	88%	–	–	–	A supervised learning model using gradient boosting and gene ontology was applied to predict genetic biomarkers of ASD, achieving high classification accuracy, demonstrating the potential of AI in genomic autism research
Ismail et al. [13]	Stacking + SMOTE + ensemble models	ASD (genetic biomarkers)	95.5%	–	–	–	An enhanced ensemble model incorporating SMOTE and stacking techniques improved prediction of ASD-associated genes, outperforming the previous model with an accuracy of 95.5%
Carroll et al. [21]	Clinical decision support system	ADHD	–	–	–	OR: 8.0	High precision in AHDH diagnosis implementing de computerized decision aid
Carroll et al. [22]	Clinical decision support system	Developmental delay	–	–	–	OR: 15.6	The tool increased the detection rate of possible developmental delays improving early identification in at-risk children
Sahamiri et al. [19]	ANN	ASD	Higher vs. other models	–	–	–	ANN overcome other models’ data to identify patterns which allows a more accurate classification of ASD
Minissi et al. [25]	SVM with BoW, RBF kernel	ASD	85%–88%	Sensitivity: 93%, specificity: 86%	0.89–0.93	–	Intervention showed high precision in ASD detection, also the SVM multimodal revealed improvement in the classification of ASD

*AUC* area under the curve, *ANN* artificial neural network, *RFE* recursive feature elimination, *SVM* support vector machine, *CNN* convolutional neural network, *DNN* deep neuronal network, *OR* odds ratio, *ADHD* attention deficit hyperactivity disorder, *ASD* autism spectrum disorder, *SMOTE* synthetic minority oversampling technique, *H-ELM* hierarchical extreme learning machine, *ML* machine learning, *VR* virtual reality, *EDA* electrodermal activity, *AI* artificial intelligence, *BoW* bag of words, *RBF* radial basis function

### Deep neural networks and neurodevelopmental disorders diagnostic accuracy

AI methodology, such as deep learning, is an advanced type of machine learning that uses artificial neural networks to analyze large volumes of data to recognize images, text, or behavioral patterns and extract complex patterns. In the articles reviewed, it was applied in six studies, where convolutional neural networks (CNNs) were highlighted for the analysis of neuroimaging and clinical data. These tools are especially useful in the early detection of brain abnormalities in high-risk pediatric populations.

For example, *“*A multitask, multistage deep transfer learning model for the early prediction of neurodevelopment in very preterm infants*”*, which uses a multitask deep learning framework for the fusion of clinical and neuroimaging data to predict multiple neurodevelopmental abnormalities early, was highly accurate in the early prediction of neurodevelopment in preterm infants, with an ROC of 0.86 for cognitive deficits, 0.66 for language deficits, and 0.84 for motor deficits [8].

On the other hand, a study in which motor outcomes in preterm infants were predicted from very early brain diffusion, magnetic resonance images (MRI) via a deep learning CNN model revealed the efficacy of CNNs in predicting motor development in preterm infants, with a sensitivity of 70%, specificity of 74%, and an ROC of 72%. It also identifies key brain regions, such as the motor cortex and somatosensory areas, for the prediction of abnormal Neurological, Sensory, Motor, Developmental Assessment (NSMDA) scores (discriminative and predictive tests of gross and fine motor development and neurological and sensorimotor performance) [9].

### Supervised machine learning and diagnosis of autism spectrum disorder and attention deficit hyperactivity disorder

The machine learning methodology was the predominant approach in ten studies, which employed algorithms such as support vector machines (SVMs), decision trees, and extreme learning machines (ELMs) for the classification of neurodevelopmental disorder subtypes.

An outstanding example is the “Multiclass classification for the differential diagnosis of ADHD subtypes via recursive feature elimination and hierarchical extreme learning machine” [14], which implements a model based on recursive feature elimination (RFE) and hierarchical machine learning model (H-ELM) to improve the accuracy of the classification of ADHD subtypes, achieving an accuracy of 92.3%, where the AI method focuses on the most relevant features—surface area of the superior frontal lobe, cortical thickness, volume and mean surface area of the entire cortex.

Additionally, “A protocol for the diagnosis of autism spectrum disorder structured in machine learning and verbal decision analysis*”* used verbal analysis and machine learning models to improve the diagnostic accuracy of ASD, resulting in a 20% improvement in diagnostic accuracy over conventional assessments [15].

Another study, “Identification of autism spectrum disorders via deep learning and the ABIDE dataset” used an SVM to differentiate children with ASD from healthy controls, with a sensitivity of 88% and a specificity of 85% [10].

Similarly, the study “Application of supervised machine learning for behavioral biomarkers of autism spectrum disorder based on electrodermal activity and virtual reality” showed that machine learning models achieved an accuracy of over 90% in identifying biometric patterns associated with ASD [11].

Finally, recent studies have incorporated supervised ensemble strategies in ASD gene prediction. Ismail et al. (2022) developed the hybrid ensemble-based classification (HEC)-ASD model, a gradient-based ensemble learning approach that achieved 88% accuracy for predicting ASD-associated genes via functional matrices and gene ontology [12]. The same group subsequently proposed the stacking-synthetic minority oversampling technique (SMOTE) model, which integrates class balancing with supervised classifiers [such as SVM, K-nearest neighbor (k-NN), and random forest], achieving 95.5% accuracy [13]. Although these studies focused on genetic data rather than behavioral or clinical biomarkers, they demonstrated the robustness of supervised learning to improve the early diagnosis of ASD via a multidimensional approach.

### Clinical decision support systems and neurodevelopmental disorders prediction

Clinical decision support systems such as AI technologies focus on feeding medical data and providing evidence-based recommendations, thereby detecting patterns that humans may miss. In this sense, four studies were evaluated, which analyzed the usefulness of computerized tools to improve ADHD diagnosis and child development surveillance. These systems have proven to be valuable tools for facilitating clinical decision-making in the detection of neurodevelopmental disorders.

For example, the study “Use of a computerized decision aid for ADHD diagnosis: a randomized controlled trial” showed that the use of an AI-based system reduced the assessment time and improved the diagnostic accuracy of ADHD by 15% compared with standard clinical assessment [21].

Similarly, “The use of a computerized decision aid for developmental surveillance and screening” showed that the implementation of AI-based tools improved the early detection of developmental delays in at-risk children, with a sensitivity of 89% and a specificity of 83% [22].

### Biosignal analysis for the early diagnosis of neurodevelopmental disorders and virtual reality

Finally, four studies were explored in this field, which combined the use of physiological sensors and virtual reality environments with AI algorithms to detect neurobehavioral patterns associated with ASD.

Machine learning techniques, such as electrodermal activity (EDA), electroencephalography (EEG), and electrocardiographic changes (ECGs), have been applied to biosignals.

A relevant study mentioned previously, “Application of supervised machine learning for behavioral biomarkers of autism spectrum disorder based on electrodermal activity and virtual reality”, combined these methodologies to improve ASD detection through physiological and behavioral responses measured in controlled environments, achieving a sensitivity of 91% and a specificity of 85% [11].

Similarly, a study of biomarkers of autism spectrum disorder based on biosignals, virtual reality, and artificial intelligence revealed that the combination of neurophysiological signals with AI improved the classification of children with ASD by 18% compared with traditional methods [23].

### Comparative performance overview

Deep learning models such as CNNs and transfer learning have shown strong diagnostic performance in neuroimaging tasks, particularly in prematurity-related conditions (e.g., AUCs up to 0.86). Supervised machine learning models—including SVM, H-ELM, and gradient boosting—were predominantly applied in ASD and ADHD classification, achieving high precision values, some above 90%. Clinical decision support systems have demonstrated significant improvements in diagnostic utility in real-world pediatric settings, whereas biosignal-based approaches (e.g., electrodermal activity combined with virtual reality) have shown promise in noninvasive ASD screening. Notably, two recent studies extended supervised models to genetic biomarker prediction, reaching accuracies of 88% and 95.5%, highlighting AI’s potential in genomic-level ASD detection (Table 3).

## Discussion

### General strengths and findings

The studies reviewed suggest that AI has great potential in the diagnosis and monitoring of NDDs, supporting improved treatment and prevention of complications. Deep neural networks have demonstrated effectiveness in identifying neuroimaging patterns, whereas supervised machine learning models have increased the accuracy of clinical subtype classification, such as in ASD and ADHD. Clinical decision support systems have also emerged as useful tools to improve objectivity in diagnostic assessments, and biosignal-based AI approaches—such as those using electrodermal activity—represent a promising frontier in noninvasive biomarker detection.

These results are consistent with earlier research. For example, Zhou et al. emphasized the effectiveness of AI in early NDD detection by neuroimaging [33]. Navarro et al. [16] reported up to 90% accuracy in ADHD prediction [16]. The neural network models used in Santarrosa et al. improved the discrimination between ADHD patients and control subjects [17]. Raya et al. demonstrated the utility of virtual reality environments in enhancing the ecological validity of ASD evaluations [18]. Ismail et al. (2022, 2023) further demonstrated that supervised models could be effectively extended to the prediction of genetic biomarkers in ASD, achieving precisions of 88% and 95.5%, respectively [12, 13].

### Implementation barriers

Despite encouraging findings, several barriers limit the practical implementation of AI models. A critical issue is the small sample sizes used in many studies. For example, Saha et al. and Qureshi et al. relied on limited datasets, which reduces the statistical power and risks overfitting. This raises concerns about the generalizability of findings to broader populations [9, 10, 14].

In addition, most models have been validated only on retrospective or experimental datasets, not in real-world clinical environments. Only a few, such as Carroll et al. (2013, 2014), have tested decision support systems directly in clinical pediatric care, confirming improved diagnostic precision and workflow efficiency [21, 22].

Moreover, heterogeneity in model design and evaluation complicates cross-study comparisons. There is currently no consensus on which data features should be prioritized, what metrics should be used for performance reporting, or which validation strategies should be implemented. This lack of methodological uniformity affects both reproducibility and clinical translation.

### Ethical considerations

While none of the studies included in this review directly addressed ethical dimensions such as data privacy, model interpretability, or equitable access, these aspects remain essential to the responsible deployment of AI in pediatric neurodevelopmental diagnostics.

First, data privacy and security are of paramount importance when sensitive behavioral or genetic data from minors are used. The management of such information requires stringent safeguards and updated consent protocols tailored to pediatric contexts. The lack of explicit mention of these issues in the reviewed studies reveals a critical gap in current research practices.

Second, the interpretability of AI models is often overlooked. Without transparency in how results are generated, healthcare professionals may struggle to trust or validate AI-generated outputs. This lack of explainability risks both overreliance on algorithmic decisions and the potential erosion of human clinical judgment.

Another concern relates to equity and access. Although the reviewed studies applied AI models in experimental or research-focused settings, none have examined the feasibility of implementation in low-resource or rural environments. The absence of such analysis may mask underlying digital divides, where children without access to digital infrastructure are excluded from the benefits of AI-assisted diagnostics.

Finally, algorithmic bias remains a latent risk when models are trained on non-representative data. Although not addressed directly by the reviewed studies, the potential for skewed performance across socioeconomic or ethnic groups warrants further exploration.

These limitations underscore the need for future studies not only to validate AI tools in clinical settings but also to systematically assess the ethical implications of their development and deployment. Integrating bioethics, regulatory standards, and stakeholder perspectives will be crucial for building trust and ensuring equitable pediatric care.

### Future research directions

To advance the integration of AI in clinical practice, future studies must prioritize the use of multicenter, large-scale datasets that reflect the diversity of pediatric populations. Prospective validations in real healthcare environments, rather than experimental setups, are essential. Efforts should also aim to standardize methodological frameworks, encompassing input selection, performance reporting, and external validation. Interdisciplinary collaboration among clinicians, AI developers, and policy-makers will be key to achieving these objectives.

Moreover, the expansion of AI into genomic applications, as illustrated by the studies of Ismail et al. [12, 13], opens new directions for personalized medicine. The incorporation of explainable AI and cost-effectiveness assessments will further enhance the acceptability and sustainability of AI systems in pediatric NDD diagnosis.

Overall, the limitations of the reviewed studies include small and homogeneous samples, lack of validation in real-world clinical settings, and methodological heterogeneity that complicates reproducibility and cross-study comparisons. These issues underscore the importance of developing standardized frameworks and conducting multicenter validations to strengthen the evidence base for the clinical application of AI in pediatric neurodevelopmental disorders.

## Conclusions

This review underscores the growing potential of AI in supporting the diagnosis and management of NDDs. Tools such as deep neural networks, supervised learning models, and clinical decision support systems have shown notable promise in enhancing diagnostic accuracy and streamlining medical evaluations, particularly in conditions such as ADHD and ASD. Moreover, the integration of AI with biosignal analysis and virtual reality technologies suggests an emerging path toward more personalized and responsive clinical care.

Nonetheless, these advances are tempered by persistent challenges, including limited population diversity in studies, lack of algorithmic standardization, and insufficient validation in real-world clinical environments. To ensure safe and effective integration into pediatric care, future efforts must prioritize multicenter validation, standardized development protocols, and robust regulatory oversight.

Ultimately, while AI holds transformative potential in pediatric neurodevelopmental assessment, its clinical adoption must be grounded in evidence-based practices that prioritize transparency, equity, and ethical responsibility.

## Supplementary Information

Below is the link to the electronic supplementary material.

Supplementary file1 (DOCX 18 kb)

## Data Availability

No new data were created or analyzed in this study. Data sharing is not applicable to this article.
