# Supplementary material for: Artificial intelligence in diagnosis of pediatric neurodevelopmental disorders: a scoping review
Source: World J Pediatr. 2026 Jan 27;22(3):315–29. doi: 10.1007/s12519-025-00999-z (PMC13076480; doi:10.1007/s12519-025-00999-z)
Supplement: Supplementary file 1 — Supplementary file1 (DOCX 18 kb) [file 12519_2025_999_MOESM1_ESM.docx]

**Table 1 Selection criteria and search strategy**

| **Inclusion** | **Exclusion** |
| --- | --- |
| 1. Children and adolescents (0-18 years) with risk or suspicion of neurodevelopmental disorders (ASD, ADHD, intellectual disability, language disorders, etc.).  2. Use of artificial intelligence (AI) in any modality (machine learning, neural networks, predictive models, image analysis, etc.).  3. Evaluation of the diagnostic accuracy of AI (sensitivity, specificity, AUC-ROC, etc.). Identification of biomarkers or clinical patterns by AI. Impact on early detection and access to interventions.  4. Types of studies: clinical trials, observational studies, systematic reviews, meta-analyses, AI validation studies, and snowball studies.  5. Studies published in the last 25 years  6. Articles in English or Spanish with full-text access. | 1. Technologies without AI (traditional statistical analysis, clinical evaluations without AI algorithms).  2. Studies that do not report diagnostic accuracy metrics.  3. Editorials, letters to the editor, commentaries, and case studies without validation in broader populations.  4. Studies published before the established range.  5. Publications in languages other than English or Spanish.  6. Studies without access to full text. |
| **Search Strategy** | |
| **Method used** | **MeSH Terms** |
| **General search** | ("Neurodevelopmental Disorders"[Mesh] AND ("Artificial Intelligence"[Mesh] AND "Pediatrics"[Mesh]) |
| **Search by subtopic** | ("Neurodevelopmental delay" OR "Autism Spectrum Disorder"[Mesh] OR "ASD" OR "Attention Deficit Hyperactivity Disorder"[Mesh] OR "ADHD" OR "Intellectual Disabilities"[Mesh] OR "Language delay" OR "Developmental Disabilities"[Mesh] OR "Cognitive Impairment" OR "Speech Delay" OR "Learning Disabilities" OR "Neurocognitive Disorders" OR "Developmental Delay" OR "Communication Disorders" OR "Global Developmental Delay") AND ("Machine Learning"[Mesh] OR "Neural networks" OR "Deep Learning" OR "Automated diagnosis" OR "AI-based diagnosis" OR "Clinical decision support system" OR "Pattern recognition in medical diagnosis" OR "Explainable AI" OR "Natural Language Processing" OR "Big Data" OR "Computer-Aided Diagnosis" OR "Predictive Modeling" OR "Algorithmic Diagnosis" OR "Decision Support Techniques" OR "Image Recognition" OR "Speech Recognition") AND ("Child"[Mesh] OR "Pediatrics"[Mesh] OR "Infant"[Mesh] OR "Toddler" OR "Adolescent"[Mesh] OR "Preschool child" OR "Young Child" OR "School-Aged Children" OR "Teenager") |

*Own elaboration.* **ASD**; autism spectrum disorders, **ADHD**; attention deficit hyperactivity disorder, **AI**; artificial intelligence, **AUC**; area under the curve, **ROC**; receiver operating characteristic curve.
